# Detection of *Plasmodium falciparum* and *Plasmodium vivax* subclinical infection in non-endemic region: implications for blood transfusion and malaria epidemiology

**DOI:** 10.1186/1475-2875-13-224

**Published:** 2014-06-06

**Authors:** Luciana MF Maselli, Debora Levy, Gabriel Z Laporta, Aline M Monteiro, Linah A Fukuya, Maria F Ferreira-da-Cruz, Claudio T Daniel-Ribeiro, Pedro E Dorlhiac-Llacer, Maria Anice M Sallum, Sérgio P Bydlowski

**Affiliations:** 1 Laboratory of Genetics and Molecular Hematology (LIM31), University of Sao Paulo School of Medicine, Av. Dr. Enéas de Carvalho Aguiar, 155 – 1st floor - room 43, São Paulo, SP 05403-000, Brazil; 2 Fundação Pró-Sangue Hemocentro de São Paulo, São Paulo, SP 05403-000, Brazil; 3 Division of Hematology, Hospital das Clínicas from the University of Sao Paulo School of Medicine, São Paulo, SP 05403-000, Brazil; 4 Departament of Epidemiology, Faculdade de Saúde Pública, Universidade de São Paulo, São Paulo, SP 01246-904, Brazil; 5 Laboratório de Informática Médica (LIM01), University of São Paulo School of Medicine, São Paulo, SP 01246-903, Brazil; 6 Malaria Research Laboratory, Instituto Osvaldo Cruz, Rio de Janeiro, RJ 21045-900, Brazil

**Keywords:** *Plasmodium falciparum*, *Plasmodium vivax*, *S*ubclinical infection, Blood center, Transfusion, Prevalence ratio, Dynamics of malaria transmission

## Abstract

**Background:**

In Brazil, malaria is endemic in the Amazon River basin and non-endemic in the extra-Amazon region, which includes areas of São Paulo state. In this state, a number of autochthonous cases of malaria occur annually, and the prevalence of subclinical infection is unknown. Asymptomatic infections may remain undetected, maintaining transmission of the pathogen, including by blood transfusion. In these report it has been described subclinical *Plasmodium* infection in blood donors from a blood transfusion centre in São Paulo, Brazil.

**Methods:**

In this cross-sectional study, representative samples of blood were obtained from 1,108 healthy blood donors at the Fundação Pró-Sangue Hemocentro de São Paulo, the main blood transfusion centre in São Paulo. Malaria exposure was defined by the home region (exposed: forest region; non-exposed: non-forest region). Real-time PCR was used to detect *Plasmodium falciparum* and *Plasmodium vivax*. Subclinical malaria cases were geo-referenced.

**Results:**

Eighty-four (7.41%) blood donors tested positive for *Plasmodium*; 57 of these were infected by *P. falciparum*, 25 by *P. vivax*, and 2 by both. The prevalence of *P. falciparum* and *P. vivax* was 5.14 and 2.26, respectively. The overall prevalence ratio (PR) was 3.23 (95% confidence interval (CI) 2.03, 5.13); *P. falciparum* PR was 16.11 (95% CI 5.87, 44.21) and *P. vivax* PR was 0.47 (95% CI 0.2, 1.12). *Plasmodium falciparum* subclinical malaria infection in the Atlantic Forest domain was present in the mountain regions while *P. vivax* infection was observed in cities from forest-surrounded areas.

**Conclusions:**

The presence of *Plasmodium* in healthy blood donors from a region known as non-endemic, which is important in the context of transfusion biosafety, was described. Infected recipients may become asymptomatic carriers and a reservoir for parasites, maintaining their transmission. Furthermore, *P. falciparum* PR was positively associated with the forest environment, and *P. vivax* was associated with forest fragmentation.

## Background

In Brazil, human malaria is mainly caused by *Plasmodium vivax*, which accounts for more than 80% of identified cases; *Plasmodium falciparum* is responsible for 16.3%, and *Plasmodium malariae* is associated with a small number of cases [1,2]. The majority of malaria is reported in the Brazilian Amazon region [3] where *Anopheles darlingi* is the primary vector in recently deforested areas [4].

The annual parasite index is typically employed to estimate the risk of occurrence of malaria in human populations that are exposed to infective *Anopheles* mosquito bites, in a specific time frame. In endemic regions, this index is spatially and temporarily variable, and its values can be low (0.1–9.9), moderate (10.0–49.9), or high (>50) [3], depending on ecological and environmental determinants that represent distinct risk in different social and economic contexts [5]. Areas of high endemicity have an elevated prevalence of individuals with subclinical infections [6]. In the Amazon region of Brazil, studies performed in Amerindians [7] and in riverine communities [8] in the state of Rondônia have shown the presence of long-term subclinical infections. Those individuals were infected with *Plasmodium* spp; however, most of them showed low levels of parasitaemia [9]. Subclinical infections also have been found in meso- and hypo-endemic regions [8,10,11]. The São Paulo municipality and nearby areas in southeastern Brazil are considered non-endemic regions; however, in 2013, 146 autochthonous cases of malaria were identified in São Paulo State, representing 22% of the total occurring outside the Amazon region. Moreover, there was a 256% increase compared to 2006 [12].

*Plasmodium* pathogens that cause malaria in humans are naturally transmitted by infectious *Anopheles* mosquito bites [13]. However, the protozoan can be accidentally transmitted by blood transfusion [14] and intravenous drug users [10]. The first case of transfusional malaria was registered in 1911 [15-17]. Although uncommon, transfusional malaria can have serious clinical implications when undetected and not treated early [18,19]. In the Amazon State, 0.3% of screened blood donors were found to be infected with *P. vivax*[20] when the diagnoses were based on an especially sensitive molecular probe, i.e., PCR technology. In Brazil, the laws governing the screening obligation for *Plasmodium* infection in blood donors from an endemic region are distinct from those adopted in non-endemic areas [21]. Furthermore, in non-endemic areas, all candidates for blood donation are subjected to an interview before they donate blood. Following the interview, potential blood donors can be either rejected or accepted for donation, without enforcement for a laboratorial screening for *Plasmodium* infection [21]. Considering both the circulation of *Plasmodium* spp. in non-endemic areas and the fact that there is no obligation for screening for *Plasmodium*, it is plausible to suppose that accidental transfusional *Plasmodium* transmission can occur, mainly by the presence of subclinical infection in humans.

Transfusion-transmitted *Plasmodium* parasites represent an important risk factor because they can cause severe malaria with a high fatality rate [22]. The chronic infection can persist in humans for a long period of time: up to two years or more for *P. falciparum*, up to seven years for *P. vivax*, and even for an entire lifetime for *P. malariae*[1]*.* Consequently, an asymptomatic human who is unaware of his/her status as a *Plasmodium* reservoir can transmit the parasites when donating blood. Usually, parasite concentration is low in asymptomatic individuals [8]; however, asymptomatic humans can both infect *Anopheles* females [9] and cause severe infection in blood recipients [22].

Employing a high sensitivity real-time PCR in a cross-sectional design, it was addressed 1) the prevalence of *P. falciparum* and *P. vivax* among blood donors at a public blood bank in the municipality of São Paulo, Brazil; 2) the spatial distribution of the blood donors; and 3) the potential association between the geographical origins of donors and the *Plasmodium* species.

## Methods

### Participants and samples

A cross-sectional study was adopted with the main objective of estimating the prevalence of subclinical malarial infections among blood donors in the State of São Paulo. The studied population was composed of 1,108 blood donors enrolled at the Fundação Pró-Sangue Hemocentro de São Paulo, Brazil, from August 2008 to March 2010. The sampling procedure employed a non-probabilistic cumulative method. Accordingly, individuals who came to donate blood were cumulatively selected regarding free potentially past/recent malarial infections as inclusion criteria. Each blood donor volunteer was interviewed for previous malaria, location of residence, and travel/visit to endemic regions. Brazilian guidelines recommend refusing as a potential blood donor any individual who has lived in a malaria-endemic region within the past three years as well as those who have travelled to those areas in the past six months. Consequently, the present study does not include individuals who were rejected for blood donation based on these conditions. In terms of sampling procedure, it was knew who would be next to donate blood nor who would be included in the study. This circumstance made the sampling procedure random, which is a reasonable proxy for obtaining a representative sample of blood donors from the population in the State of São Paulo [23].

To calculate the sample size, the prior prevalence of subclinical malarial infections among blood donors was considered to be 1%. This subclinical malaria information is unavailable for the State of São Paulo, and the sample size for the cross-sectional study thus was estimated using *Plasmodium* spp. prevalence (1.3%) in blood banks in the State of Amazon [3]. Considering that prevalence was presumed as 0.01, standard error was tolerable at 0.02, and 95% confidence intervals were selected and applied. The following statistical equation of minimum sample size calculation for prevalence data was used:

$$n^{*}=z_{\frac{\alpha }{2}}^{2}\frac{\sqrt{P\left(1-P\right)}}{\epsilon^{2}}$$

where n* = minimum sample size; *z* = critical percentile of the z distribution (1.96); *α* = significance level (0.05); *ϵ* = standard error or tolerable error (0.02 or 2%), which measures discrepancy between population prevalence (unknown) and estimated prevalence from the obtained sample; and *P* = prior prevalence data (as supported by [24-26]). Based on the values obtained in the sample size estimation, the study had to include blood from at least 956 individuals to estimate the prevalence of malarial infections among blood donors with a confidence interval of 95% and precision of 2%.

**Figure 1:**
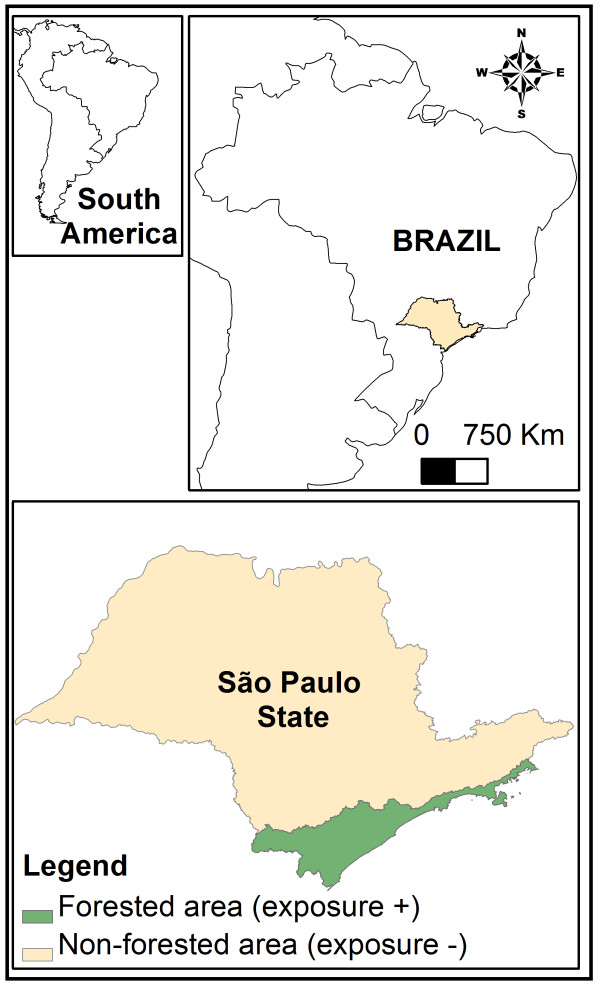
**Study System: South America, Brazil, São Paulo State.** Exposure status was defined by considering the presence/absence of forested regions in the State of São Paulo. Forested regions are localized in the southeast of the State of São Paulo and represent remnants of the Atlantic rainforest.

Once the representative sample (N) was obtained, four groups were defined: disease-exposed (a), non-disease exposed (b), disease non-exposed (c), and non-disease non-exposed (d). Exposure status was defined based on evidence of risk of being in contact with infectious mosquito bites (e.g., location of residence that may indicate the presence of *Anopheles* mosquito vectors). Individuals living in forested areas of the Atlantic Forest were considered at risk of infection whereas those from urban areas in the Metropolitan Region of São Paulo were considered to be without risk of malaria (Figure 1). No individual had fever or evident signs of infection at the time of the blood donation; thus, disease and non-disease status were evaluated using real-time PCR. Peripheral venous blood (5 mL) was drawn from the volunteers and screened for *P. falciparum* and *P. vivax* by real-time PCR.

Each volunteer was informed about the research and research procedures before signing written informed consent. Both protocol and procedures employed in the present study were approved by the Research Ethics Committee of the institution.

### Plasmodium identification by real-time PCR

DNA was extracted from blood samples using a salting-out method with slight modifications [27,28]. DNA integrity was analysed by agarose gel electrophoresis, and the concentration was determined with a nanoDrop ND-1000 spectrophotometer (USA) [29]. Samples of *P. falciparum* and *P. vivax* were used as positive control for the real-time PCR protocol.

RT-PCR was performed following the protocol described by Gama *et al.*[30]. Two-hundred ng of DNA were added to a 12.5 μL reaction mixture containing 1X TaqMan Universal PCR Master Mix (Applied Biosystems, Foster City, CA, USA) with 500 mM of each dNTP. The primers and probe sequences employed are listed in Additional file 1. Each sample was analysed in duplicate. Real-time PCR amplification was performed in a Rotor Gene 3000 (Corbett Research, San Francisco, CA, USA) using a program involving two thermal cycler holds (50°C for 2 min and 95°C for 10 min), followed by 45 cycles of amplification (95°C for 15 s, 52°C for 60 s, and 72°C for 60 s). Results were automatically analysed using real-time Rotor Gene 6 software.

The efficiency and sensitivity of the real-time PCR protocol were examined using genomic DNA extracted from lab-cultured *P. falciparum* parasites, and *P. vivax* from the blood of humans with a confirmed diagnosis of vivax malaria. A standard curve was constructed for each *Plasmodium* species from a 10-fold serial dilution of parasite DNA, ranging from 10^6^ to 0.1 copies/μL. The threshold limits were automatically set for each reaction, and mean Ct was calculated for each amplified triplicate. Amplification efficiencies for the different primer pairs and probes were calculated with the following formula: Efficiency = 10(−1/Slope) − 1 (Additional file 1). Samples were considered positive when the amplification curves reached the threshold line. A positive PCR assay was demonstrated at 0.00001% parasitaemia (0.5 parasite/μL) for all *Plasmodium* species.

To evaluate the intra- and inter-assay Ct variation of real-time PCR, we analyzed the coefficient of variation of DNA parasite samples in triplicate and in the blood samples showing low parasitaemia. Pure parasite DNA showed a Ct coefficient of 0.1 while the Ct coefficient was 0.3 among low-parasitized blood samples. The Ct values were the same and stable for each DNA parasite sample in different experiments.

No cross-amplification between different reactions was observed. A PCR amplification product was obtained only when specific *Plasmodium* species were present in the reaction; no PCR product was detected in samples without specific parasites.

### Data analysis

In this cross-sectional study, the starting point was a representative sample (N) obtained for defining the four groups under study (a, b, c, d). First, the prevalence of *Plasmodium* infections (*P. vivax* and *P. falciparum*) was estimated, using the following equations:

$$P_{t}=\frac{a_{t}+c_{t}}{N};P_{\mathrm{pv}}=\frac{a_{\mathrm{pv}}+c_{\mathrm{pv}}}{N};P_{\mathrm{pf}}=\frac{a_{\mathrm{pf}}+c_{\mathrm{pf}}}{N}$$

where a + c is the number of individuals carrying the malaria parasite, and t = all parasites, pv = *P. vivax*, and pf = *P. falciparum*. N is the representative sample. The prevalences (P_t_, P_pv_, P_pf_) represented estimates of the magnitude of the malarial subclinical infections on the blood donor population. Then the PR was estimated, giving a measure of the prevalence of subclinical malarial infections in the exposed group in relation to the non-exposed group, using the following equation:

$$PR_{t,\mathrm{pv},\mathrm{pf}}=\frac{\frac{a_{t,\mathrm{pv},\mathrm{pf}}}{a_{t,\mathrm{pv},\mathrm{pf}}+b_{t,\mathrm{pv},\mathrm{pf}}}}{\frac{c_{t,\mathrm{pv},\mathrm{pf}}}{c_{t,\mathrm{pv},\mathrm{pf}}+d_{t,\mathrm{pv},\mathrm{pf}}}}$$

where a = the number of subclinical malarial infections in the exposed group; c = the number of subclinical malarial infections in the non-exposed group; a + b = total exposed individuals; c + d = total non-exposed individuals. PR was estimated for each parasite, with t = all parasites, pv = *P. vivax*, and pf = *P. falciparum*. If the presence of a certain exposure factor (e.g., forest proximity) does not increase the prevalence of subclinical malaria, the PR is expected to be 1; PR > 1 indicates a positive association between exposure and prevalence; and a PR < 1 suggests that the association is negative. A confidence interval of 95% was provided for each estimate of the PR. Additional theoretical rationale for these procedures can be obtained from Rothman [31].

The detected subclinical *Plasmodium* infection cases were geo-referenced using resident address and routines implemented in the R 3.0.0 programming environment with the aid of the ggmap package [32]. With this routine, addresses in a specific format (e.g., “100 Winter Avenue, Dream City NY”) were searched in the databases of Google Maps^TM^. These searches returned geographic coordinates (longitude and latitude) that were plotted over a satellite imagery scenario provided by Google Maps^TM^. Geo-referenced data were used to identify possible explanations considering the observed pattern of geographic distributions of subclinical malarial infections.

## Results

A total of 1,108 individuals were selected in the present study. From this sample, 61 were exposed and had a subclinical malaria infection; 439 were exposed and did not have a subclinical malaria infection; 23 were non-exposed and had a subclinical malaria infection; and 585 were non-exposed and did not have a subclinical infection (Table 1). The overall prevalence of subclinical malarial infections among blood donors was 7.58 (%); prevalence among exposed individuals was 12.20 (%) and among non-exposed, it was 3.78 (%) (Table 1). The PR was 3.23 and showed a positive statistically significant association between exposure and subclinical malarial infection (CI 95% = 2.03, 5.13) (Table 1).

**Table 1:** Distribution of individual blood donors according to exposure status and subclinical malarial infections in a cross-sectional study design, São Paulo State, Brazil, Aug 2008–Mar 2010

	Disease (+)*	**Disease (−)**	**Total**	Prevalence**
Exposed (+)	61	439	500	12.20
Exposed (−)	23	585	608	3.78
Total	84	1,024	1,108	7.58

*In each group (exposed and non-exposed), 1 subject was infected by both parasites.

**Cases per 100 population units.

Prevalence ratio (95% CI) = 3.23 (2.03, 5.13).

Of the subclinical malarial infections (84), 57 were *P. falciparum*, 25 were *P. vivax*, and two were mixed (*P. vivax* and *P. falciparum*). Considering only *P. falciparum* subclinical infections, 53 blood donors of the exposed group were positive for subclinical infection, 447 were exposed and did not have any evidence of infection, 4 individuals of the non-exposed group tested positive, and 604 tested negative for infection (Table 2). The total prevalence of subclinical *P. falciparum* infections in blood donors was 5.14 (%); the prevalence among exposed individuals was 10.60 (%); and among non-exposed, it was 0.66 (%) (Table 2). The prevalence ratio was 16.11 and showed a positive, statistically significant association between exposure and subclinical *P. falciparum* infection (CI 95% = 5.87, 44.21) (Table 2).

**Table 2:** **Distribution of individual blood donors according to exposure status and *P*. *falciparum *** **subclinical infections in a cross-sectional study design, São Paulo State, Brazil, Aug 2008–Mar 2010**

	**Disease (+)**	**Disease (−)**	**Total**	Prevalence*
Exposed (+)	53	447	500	10.60
Exposed (−)	4	604	608	0.66
Total	57	1,051	1,108	5.14

*Cases per 100 population units.

Prevalence ratio (95% CI) = 16.11 (5.87, 44.21).

Considering the analysis of *Plasmodium vivax* subclinical infections, seven exposed participants had a subclinical infection, 493 were exposed without subclinical infection, 18 non-exposed had subclinical infection, and 590 non-exposed did not have subclinical infection (Table 3). The total prevalence of subclinical *P. vivax* infection among blood donors was 2.26 (%); prevalence among exposed individuals was 1.40 (%), and among non-exposed, it was 2.96 (%) (Table 3). The PR was 0.47 and showed a negative statistically non-significant association between exposure and subclinical *P. vivax* infection (CI 95% = 0.2, 1.12) (Table 3).

**Table 3:** **Distribution of individuals blood donors according to exposure status and *P*. *vivax *** **subclinical infections in a cross-sectional study design, São Paulo State, Brazil, Aug 2008–Mar 2010**

	**Disease (+)**	**Disease (−)**	**Total**	Prevalence*
Exposed (+)	7	493	500	1.40
Exposed (−)	18	590	608	2.96
Total	25	1,083	1,108	2.26

*Cases per 100 population units.

Prevalence ratio (95% CI) = 0.47 (0.2, 1.12).

**Figure 2:**
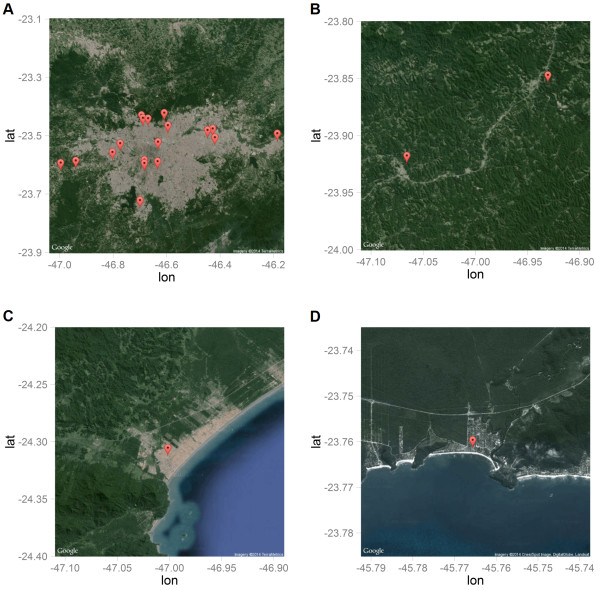
**Subclinical malarial infections geo-referenced.** Point XY coordinates of the residence of positive individuals and the presence of parasites. **A)** Presence of *P. vivax* subclinical infections in the São Paulo Metropolitan Region. **B)** Presence of *P. falciparum* subclinical infections in the highlands of the Atlantic Forest (Juquitiba and São Lourenço da Serra). **C)** Presence of *P. vivax* subclinical infection in the coastal city of Peruíbe. **D)** Presence of *P. vivax* subclinical infection in the beaches of Praia do Una (São Sebastião). Images are from GoogleMaps^TM^.

The 18 individuals with *P. vivax* subclinical infection in the non-exposed group (Table 3) were geo-referenced. These data showed that the majority of participants lived in residences near the forest fringes (Figure 1A), although these residences were in an urban area, i.e., the São Paulo Metropolitan Region. Residents infected with *P. falciparum* in the exposed region (Atlantic Forest) were from the mountains (mainly in the Juquitiba and São Lourenço da Serra municipalities). In both municipalities, forest cover is greater than 50%, and landscape fragmentation provides high levels of interaction between the forest and the urban, anthropogenic environment (Figure 2B). Individuals infected with *P. vivax* in the exposed region were from the coastal region of the Peruíbe (Figure 2C) and São Sebastião (Figure 2D) municipalities. These individuals inhabit houses located at the frontier of the Atlantic Forest and the urban landscape, which form a mosaic of human-modified and natural environments that facilitate contact between humans and sylvatic mosquitoes (Figures 2C and D).

## Discussion

Despite control and prevention measures, malaria has not been eliminated from non-endemic areas in southeastern Brazil. Autochthonous cases have been identified in several regions [33-35], and human malaria is predominantly caused by *P. vivax* and its variants [36,37]. Subclinical *Plasmodium* infections have been considered rare in Brazil; however, they have been detected and identified in endemic regions [38]. A retrospective analysis of human malaria in the State of São Paulo from 1985–2006 found 83 cases, four of which were subclinical infections [12]. Other studies have shown a low parasitaemia rate in humans who inhabit areas within the Atlantic Forest, with cases caused by *P. vivax* and *P. malariae*[36].

Recently, molecular tools have been employed to detect *Plasmodium* infection in human blood. Although PCR techniques do not represent a rapid test for malaria diagnosis, they allow detection of as little as 0.5–1 parasite/μL blood, making them 20 times more sensitive than thick blood smear microscopic examination [30]. PCR methods are also more sensitive in verifying mixed infections [39]. Additionally, real-time PCR allows pathogen quantification, reduces the risk of cross-contamination, provides accurate results [40], and identifies subclinical infections in humans [37] that might otherwise remain undetected by routine tests for malaria. Consequently, the new technology reveals a prevalence of subclinical infections in humans that is higher than previously thought [41].

Human reservoirs can maintain transmission in areas of low, moderate, or high endemicity because they can infect mosquitoes [42]. Despite the role of subclinical infection in maintaining malaria transmission, little is known about the factors that determine its occurrence and whether it is associated with human protective immune responses [41].

Blood transfusion is an important intervention in hospitals and health centres, but it also represents a route for transmission of parasites and pathogens that circulate in the blood, such as HIV, human *Plasmodium*, viruses, and others [43]. Consequently, several measures are being taken to improve blood recipient safety, including high-sensitivity screening tests. Considering only *Plasmodium*, after the collection of the blood, the parasite can survive for at least one week at 4°C, as well as in frozen erythrocytes [44]. *Plasmodium* transmission by transfusion usually occurs through whole blood and red blood cells and may occur less frequently through platelet concentrates, white blood cells, cryoprecipitate, and fresh frozen plasma [18,45]. The blood bank is therefore an important target for controlling undesirable *Plasmodium* transmission in endemic [38,46] and non-endemic regions [43,47].

Here, subclinical malarial infections were detected from blood donors in the most important centre of public healthcare in the State of São Paulo. The presence of *P. falciparum* among blood donors and the high prevalence of these infections were not expected. In the present study, each blood donor was interviewed and denied having been in malaria endemic regions. Consequently, it is plausible to assume that all subclinical infections were autochthonous. It is noteworthy that all individuals reported here would be accepted for blood donation using the current protocol adopted in blood transfusion centres in the State of São Paulo, which includes a questionnaire and an interview to detect history of exposure to malaria, both applied by a qualified professional. Therefore, although the clinical importance of the findings described here remains to be investigated, the presence of asymptomatic *Plasmodium* infection in blood donors in non-endemic areas should be an important concern for medical services, health authorities, and blood recipient safety [14].

*Plasmodium vivax* and its variants as well as *P. falciparum* and *P. malariae* circulate in non-endemic areas, where they are responsible for only a few, mostly oligosymptomatic cases of malaria annually [1]. Because *Plasmodium* species can circulate in cycles that involve humans with low parasitaemia and sylvatic mosquitoes, and likely in non-human primate reservoirs [48,49], malaria can be maintained indefinitely in silent cycles without the consequences of high-level epidemics. The estimated PR of subclinical infection found in blood donors in São Paulo clearly shows that *P. falciparum* is positively associated with forested areas. Juquitiba (30,239 inhabitants, 55.03 inhabitants/km^2^) and São Lourenço da Serra (14,874 inhabitants, 75 inhabitants/km^2^) are primarily rural locations embedded in a well-preserved and extensively forested area on the slope of Serra do Mar, where the vectors are mainly sylvatic mosquitoes from the subgenus *Kerteszia* of *Anopheles*. The human population from these areas was here defined as the exposed population whereas inhabitants of urban areas intercalated by forest fragments formed the non-exposed population. In the non-exposed population, the PR of *P. vivax* revealed a positive (PR = 0.47; 95% CI, 0.2, 1.12) between the parasite infection and urban inhabitants. However, this result may arise from the fact that the residences of the blood donors are situated in urban areas at the edge of forest fragments. Consequently, the presence of *P. vivax* is likely to be associated with forest fragments and involves both *Kerteszia* and *Nyssorhynchus* species, or even other *Anopheles* subgenera [48,50]. The evolution of transmission of *Plasmodium* species that cause malaria in humans is a dynamic process that involves ecological, environmental, and climate factors as well as social, political, and economic determinants. The high prevalence of *Plasmodium* spp. subclinical infections in blood donors living either in forested areas or urban regions intermixed with forest fragments suggests that some evolutionary mechanisms are modulating the virulence of *P. falciparum* and *P. vivax* in a way that does not negatively affect survival of the parasite, humans, mosquitoes, and non-human primates that seem to be involved in malaria transmission in the Atlantic Forest. The dynamics of human malaria transmission in the Brazilian Amazon forest seems to involve similar evolutionary mechanisms that lead to subclinical infections in humans [42], and transmission cycles that involve humans and other mosquito species as vectors (in addition to *An. darlingi*) [51], and non-human primates, which were recently found to be infected with *P. falciparum*[52]. In the Atlantic Forest, *Alouatta* monkeys have been found to be infected by *P. vivax* and *P. malariae*[49], along with *P. falciparum*[48].

The scientific novelty and translational importance of the present results are two-fold: 1) They could represent a serious problem for the blood transfusion programme in the State of São Paulo; and 2) they could be evidence of evolutionary mechanisms of attenuation of malarial symptoms in humans.

The translational importance of the first aspect is obvious. Because some infected recipients may become asymptomatic carriers and constitute a reservoir for parasites, maintaining their transmission, further investigations are needed to better document the risk of transfusion-transmitted malaria in these regions and to establish whether a specific malarial infection test should be considered for blood donors who live in or near the Atlantic Forest. The second aspect is more subtle and needs more data before being established as a paradigmatic cornerstone for the evolution of malaria transmission dynamics. In terms of clinical and economic viewpoints, the Atlantic Forest’s dynamics of malaria transmission could be a real-world example of symptomatic malaria elimination.

## Conclusions

The presence of subclinical *P. falciparum* and *P. vivax* infection in healthy blood donors from São Paulo a non-endemic region was described. This finding is a significant concern in the context of transfusion biosafety. Some infected recipients may be asymptomatic carriers, constituting a reservoir for parasites, maintaining their transmission and increasing the risk of transfusion-transmitted malaria in these regions. Additionally, prevalence ratio infection values showed a positive association between forest environment and *P. falciparum* infection, which was linked to residence in the mountain region of the Atlantic Forest domain. Moreover, *P. vivax* seemed to be associated with forest fragmentation because it was identified in residents of cities in forest-surrounded areas. These results indicate a link between the pathogen prevalence and the Atlantic Forest environment.

## Competing interests

The authors declared that they have no competing interests.

## Authors’ contribution

AMM, PEDL and SPB conceived the idea. LMFM, DL, GZL, AMM, MAMS and SPB participated in the study design and drafted the manuscript. AMM and PDLC supervised sample collection. LMFM, DL, GZL, LAF, MFFC and CTDR performed the experiments. LMFM, DL, GZL, AMM, CTDR, PEDL, MAMS and SPB analysed and interpreted the data. All authors read and approved the final version of the manuscript.

## Supplementary Material

Additional file 1 **Primers and probes used for detection of *P. falciparum *** **and *P. vivax *** **by real-time PCR.** Description: The data provided the primers and probes used to perform real-time PCR. Click here for file
