# Supplementary material for: Detection of Plasmodium falciparum and Plasmodium vivax subclinical infection in non-endemic region: implications for blood transfusion and malaria epidemiology
Source: Malar J. 2014 Jun 6;13:224. doi: 10.1186/1475-2875-13-224 (PMC4059091; doi:10.1186/1475-2875-13-224)
Supplement: Additional file 1 — Primers and probes used for detection of P. falciparum and P. vivax by real-time PCR. Description: The data provided the primers and probes used to perform real-time PCR. [file 1475-2875-13-224-S1.doc]

**Table S1.** Primers and probes used for detection of *P. falciparum* and *P. vivax* by real-time PCR

| **Species** | **Primers and probes** | **Efficiency (%)** | |
| --- | --- | --- | --- |
| *P. falciparum* | FAL-F CTTTTGAGAGTTTTGTTACTTTGAGTAA  FAL-R TATTCCATGCTGTAGTATTCAAACACAA  FAL probe TGTTCATAACAGACGGGTAGTCATGATTGAGTTCA | | 105 |
| *P. vivax* | VIV-F ACGCTTCTAGCTTAATCCACATAACT  VIV-R ATTTACTCAAAAGTAACAAGGACTTCCAAGC  VIV probe TTCGTATCGACTTTGTGCGCATTTTGC | 102 | |
